# C_f_/SiC Ceramic Matrix Composites with Extraordinary Thermomechanical Properties up to 2000 °C

**DOI:** 10.3390/nano14010072

**Published:** 2023-12-26

**Authors:** Min-sung Park, Jian Gu, Heesoo Lee, Sea-Hoon Lee, Lun Feng, William G. Fahrenholtz

**Affiliations:** 1 Extreme Materials Institute, Korea Institute of Materials Science, Changwon-si 51508, Republic of Korea; pms800@pusan.ac.kr; 2 School of Materials Science and Engineering, Pusan National University, Busan 46241, Republic of Korea; 3 College of Materials Science and Engineering, Nanjing Technical University, Nanjing 210037, China; 202210007346@njtech.edu.cn; 4 Materials Research Center, Missouri University of Science and Technology, Rolla, MO 65409, USA; allen13521@gmail.com (L.F.); billf@mst.edu (W.G.F.)

**Keywords:** silicon carbide (SiC), ceramic matrix composite (CMC), spark plasma sintering method (SPS), precursor impregnation and pyrolysis (PIP)

## Abstract

The thermomechanical properties of carbon fiber reinforced silicon carbide ceramic matrix composites (C_f_/SiC CMCs) were studied up to 2000 °C using high-temperature in situ flexural testing in argon. The CMC specimens were fabricated using an ultrahigh concentration (66 vol%) aqueous slurry containing nano-sized silicon carbide powder. The SiC powder compacts were obtained by drying the slurry and were densified using the precursor impregnation and pyrolysis (PIP) method with field assisted sintering technology/spark plasma sintering (FAST/SPS). The high relative density of the SiC green body (77.6%) enabled densification within 2.5 days using four PIP cycles. In contrast, conventional PIP processes take over 7 days. The in situ flexural strength of the C_f_/SiC CMC was 434 MPa at 1750 °C, which was 84% higher than the room temperature value. The value further increased to 542 MPa at 2000 °C. Possible mechanisms to explain the excellent strength of the CMC at elevated temperatures are discussed.

## 1. Introduction

Silicon carbide (SiC) is well known for its excellent chemical stability and mechanical properties at elevated temperature. Some of the favorable properties are its high hardness, strength, and wear resistance, low coefficient of thermal expansion, high decomposition temperature (around 3100 K), and excellent oxidation resistance. Therefore, SiC-based materials have been studied and used for a wide range of harsh environment applications, such as grinding media, brake linings, heating elements, power electronic devices, and high-temperature structures, including turbines, aerospace, and nuclear energy systems [1,2,3,4]. The ceramic matrix composites (CMCs) used for high-temperature structures are high-tech and high-value applications of SiC-based products, for instance, turbine engine components. In order to improve the fuel efficiency and trust-to-mass ratio of aircraft engines, the turbine inlet temperature (TIT) needs to be higher than the capabilities of superalloys and TBCs. International collaboration and research programs that started in the 1990’s resulted in successful commercial application of SiC-based CMCs in military and commercial aircraft engines with high TIT and pressure, such as in the General Electric/Rolls-Royce F136 engine and the CFM LEAP engine [1,4,5]. In the case of high-temperature structures for inert environments, rocket motor nozzles, missile nose cones, and other aerospace structures, such as leading-edge hyper-sonic vehicles, are potential applications for carbon fiber reinforced silicon carbide (C_f_/SiC) CMCs [6,7].

Silicon carbide is also known to be a brittle and difficult-to-sinter material, which are due to the extra-strong covalent bonding between Si and C [3,8,9]. To overcome the brittleness and enhance the mechanical properties, numerous studies have been performed to produce CMCs through the use of carbon or silicon carbide fibers [10]. Carbon fibers have superior high-temperature tolerance. On the other hand, SiC fiber has the advantages of excellent oxidation resistance and the same coefficient of thermal expansion as the SiC matrix. However, the properties of SiC fibers do not preclude the use of C_f_/SiC CMCs in oxidizing atmospheres. To overcome the low sinterability, sintering additives or nonconventional sintering methods such as field-assisted sintering technology/spark plasma sintering (FAST/SPS) or hot pressing have been applied [1,3,8,9,11,12,13]. Previous studies have shown that melt infiltration (MI) followed by hot pressing decreases the thermal stability of the matrix due to the presence of residual silicon or sintering additives. In the case of chemical vapor infiltration (CVI), uniform and effective infiltration of the matrix by a gas phase route enables excellent properties and near net-shaped CMC production at relatively low temperatures [4,14], but long processing times and high costs have suppressed the wide use of CVI [10,15].

Another method used for SiC densification is the precursor impregnation and pyrolysis (PIP) process, which uses a liquid precursor that transforms into SiC during pyrolysis. However, this process requires effective impregnation (infiltration) of the precursor and repeated PIP cycles to fabricate dense materials because of the mass loss and volume reduction during pyrolysis. In addition, the high temperature stability of the precursor-derived ceramics (PDCs) is not as robust as that of conventional ceramics because the crystallization of amorphous PDC at 1400 °C or above induces shrinkage and crack formation, which reduce the strength of the materials. To overcome the problems of PIP, the present authors conducted research involving fabricating high-density green bodies using an aqueous slurry with an ultrahigh concentration of solids and optimized each step of the PIP process [12,13]. High-purity SiC, which was expected to exhibit excellent high-temperature properties, was used as a filler, and a liquid SiC precursor was used. Unlike MI or hot pressing, no residual phases are formed that might deteriorate the elevated properties.

In this study, an aqueous slurry containing 66 vol% solids infiltrated the carbon fibers. Tape casting was used to form green tapes that were then stacked, followed by densification using the PIP method and spark plasma sintering (SPS). The elevated temperature strength of the resulting C_f_/SiC CMC was tested up to 2000 °C in argon. Possible mechanisms are suggested for retention of the elevated temperature strength.

## 2. Materials and Methods

SiC filler powder (d50: 170 nm, specific surface area: 15.8 m^2^/g) was synthesized from silicon (99.4%, 7 μm, Zhejiang Kaihua Yuantong Silicon Industry, Jiande, Zhejiang, China) and carbon black (99.9%, Alfa Aesar, Haverhill, MA, USA) powders. The starting materials were mixed in dry form in the stoichiometric molar ratio of SiC by mechanical alloying using a planetary mill (Pulverisette 5/4, Fritsch, Idar-Oberstein, Germany). Details of the powder synthesis method are described elsewhere in detail [12,13]. Fine nano-scale powder was acquired by removing most of the coarse particles that were over 1 μm in diameter by sedimentation in ethanol. SiC particles were initially preheated to 800 °C for 30 min in air to promote surface oxidation to enhance the dispersion behavior. Oxidation induces the formation of glassy SiO_2_ layer on the surfaces of SiC particles, which traps metallic ions at the surface and suppresses their spreading into the dispersoid. The metallic ions lead to the compression of the electrical double layer in the aqueous suspension and deteriorate the dispersion stability. The aqueous slurry was prepared from the SiC filler powder, deionized water and polyethylene glycol (PEG) dispersant (molecular weight = 2000, Alfa Aesar, Haverhill, MA, USA), which were mixed in a 20 mL vial. A well dispersed slurry was obtained by ultrasonication using an ultrasonic liquid processor (VCX750, Sonics & Materials, Newtown, CT, USA) for 30 min. Detailed preparation of the high-concentration SiC slurry for C_f_/SiC CMC fabrication were reported in a previous study [13]. Carbon fibers (T300, Toray, Tokyo, Japan) (density: 1.8 g/cm^3^) with a 200 nm thickness of pyrocarbon (PyC) coating [6] were aligned on a polyester film for the homogeneous infiltration of the slurry by tape casting. Tape casting was performed with a well-dispersed SiC slurry, and a doctor blade was used to push the slurry through the carbon fibers attached to the film. The thickness of the green tape was determined by adjusting the blade height during the tape casting process and was either 150 or 200 μm. After spraying a polyvinyl alcohol (PVA) solution (5 wt% in a distilled water) as binder and subsequent drying, the green tapes were cut into squares of ~30 mm on each side for stacking. Stacked green tapes were heat treated using SPS system (Dr. Sinter, Sumitomo Coal Mining Co., Tokyo, Japan) at 1550 °C for 2 h in vacuum under 10 MPa uniaxial pressure to remove SiO_2_ and H_2_O before the PIP process. Removal of SiO_2_ and neck formation or bonding among the SiC filler particles were demonstrated in a previous study [13]. Commercially available allyl hydrido polycarbosilane (AHPCS, SMP-10, Starfire Systems, Glenville, NY, USA) was employed as the SiC precursor [16]. The PIP process was conducted in the following sequence: (1) preheating of the precursor to 80 °C to decrease the viscosity and promote infiltration; (2) vacuum infiltration (VI) at 10^−2^ atm pressure for 10 min after pouring the slurry into a graphite mold with the heat-treated C_f_/SiC CMC specimen; (3) cross-linking at 300 °C (heating rate 50 °C/min) for 2 h under flowing argon (Ar); (4) pyrolysis at 1500 °C (heating rate 50 °C/min) for 1 h under flowing Ar; and (5) cooling to room temperature rapidly by turning off the SPS power. The pyrolysis temperature was high enough to promote matrix densification but low enough to use cheaper heating options, such as a conventional tube furnace. All the processes were carefully designed for minimizing contact with air.

After repeating the PIP cycle four times, CMCs were machined into bars that were 1.5 mm by 2.0 mm by 30 mm for strength testing. Phases of the specimens were analyzed by Rietveld refinement (High Score Plus, Malvern PANalytical, Malvern, UK) of X-ray diffraction (XRD, D/MAX 2500; Rigaku, Tokyo, Japan) data collected using Cu-Kα radiation. Elevated temperature, in situ strength testing was conducted following ASTM C1211-18 [17] under an argon atmosphere at temperatures of 1750 °C and 2000 °C, 5 specimens per each condition. Specimens were heated in an induction furnace at 60 °C/min to 900 °C and 100 °C/min from 900 °C to the test temperature. The fractured surfaces of the specimens were observed using scanning electron microscope (SEM, JSM-6700F; JEOL, Tokyo, Japan), after applying a gold (Au) conductive coating. Further microstructure analysis was conducted by transmission electron microscope (TEM, JEM-2100; JEOL, Tokyo, Japan). Micro-computed tomography (micro-CT, Vtomex M300; Waygate Technologies, Hürth, Germany) was used to observe cracks and other defects in the specimens.

## 3. Results and Discussions

### 3.1. Specimen Preparation

**Figure 1:**
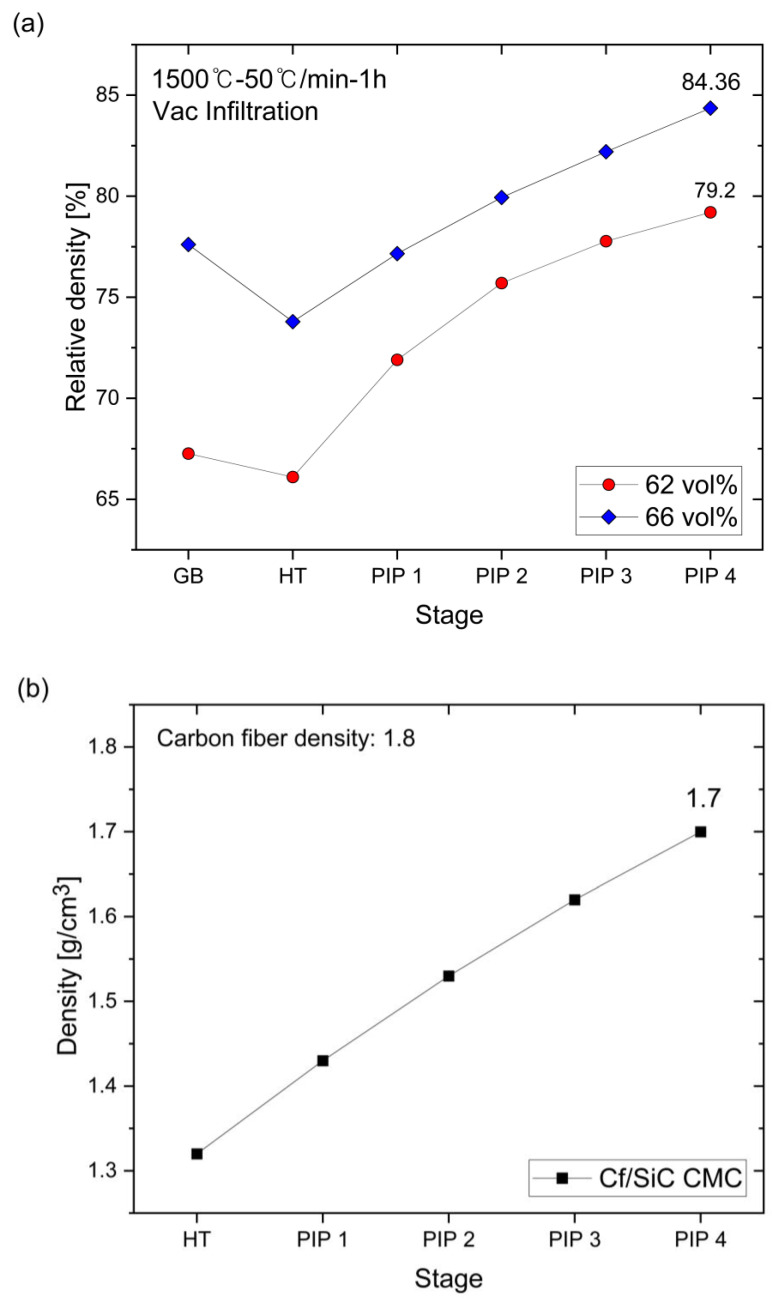
Densification behavior of (**a**) SiC_p_/SiC PRC using 62 vol% and 66 vol% slurries and (**b**) C_f_/SiC CMC by PIP process.

The densification behavior of the matrix material, a SiC_p_/SiC particulate-reinforced composite (PRC), and the C_f_/SiC CMC is presented in Figure 1. The density gradually increased after each PIP stage. The slurry with 62 vol% SiC showed a relatively rapid increase in density after the first PIP stage, and then the increase in density gradually decreased with each successive step. The relative density of the matrix prepared using the slurry containing 66 vol% increased at a consistent rate until the fourth PIP stage, where it reached 84.4% of the relative density. In comparison, the 62 vol% and 66 vol% slurry results clearly indicated that the application of a high-concentration slurry is beneficial for producing a PRC with higher relative density.

One of the disadvantages of the PIP process is the decrease in densification efficiency with the increasing the number of infiltration/pyrolysis steps. As close porosity increases, the infiltration of the liquid precursor into the specimen is suppressed [15]. However, the densification efficiency did not decrease for up to four PIP cycles for the PRC, as shown in Figure 1a, indicating that the formation of closed pores did not occur at the surface of the specimens for up to four PIP cycles.

Likewise, the density of the C_f_/SiC CMC increased for up to four PIP cycles and reached 1.7 g/cm^3^ (Figure 1b).

**Figure 2:**
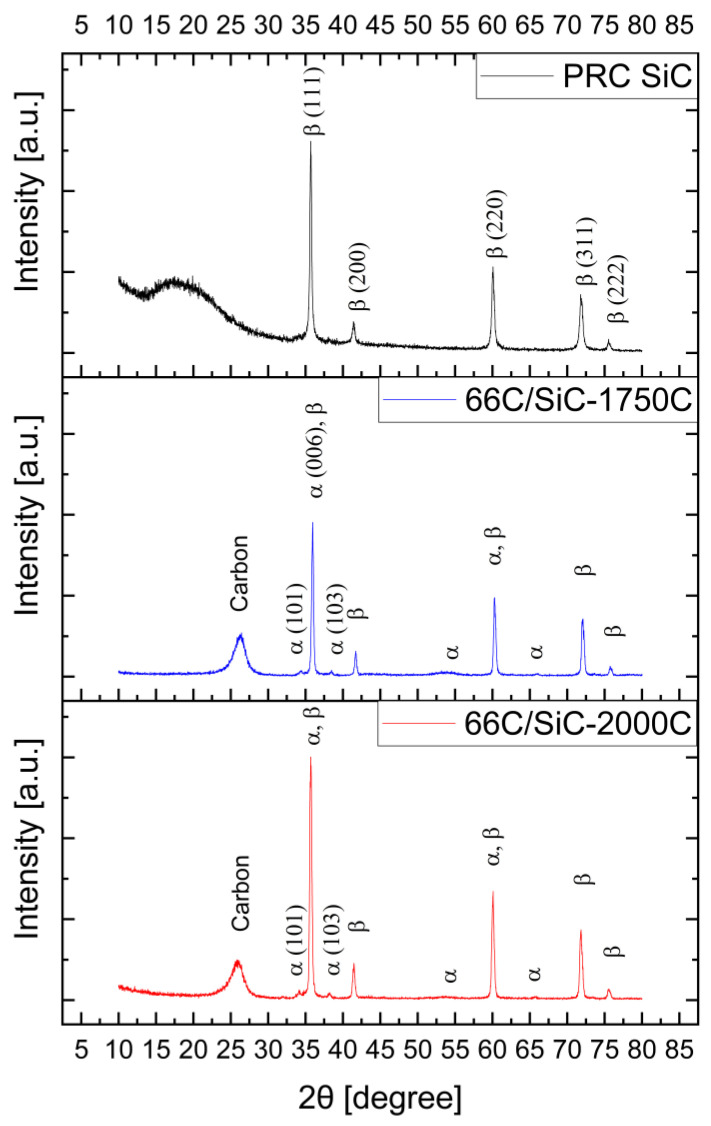
X-ray diffraction patterns of SiC_p_/SiC PRC and C_f_/SiC CMC specimens. (PRC-SiC: as-fabricated PRC, 66C/SiC-1750C: CMC after flexural testing at 1750 °C, 66C/SiC-2000C: CMC after testing at 2000 °C).

Figure 2 shows the XRD patterns of the as-fabricated SiC_p_/SiC PRC specimens. The peaks attributed to β-SiC originated from the filler and were identified along with a broad amorphous hump from the PDC that extended from 15° to 20°. A small amount of α-SiC was present in the C_f_/SiC CMC specimens, which was indicated by the (101) and other peaks of α-SiC.

### 3.2. Elevated Temperature In Situ Strength Testing

Figure 2 also shows the XRD patterns of the C_f_/SiC CMC specimens after flexural testing at 1750 °C and 2000 °C. Different from the data of the PRC SiC, a broad peak from the carbon fiber was detected near 26 degrees [18]. Although the peak matches the carbon fiber peak, it may also include the formation of graphite due to the decomposition of SiC at high temperatures. In addition, the peaks from α-SiC were identified together with those of β-SiC, indicating that phase transformation from β- to α-SiC began to occur at or below 1750 °C.

**Figure 3:**
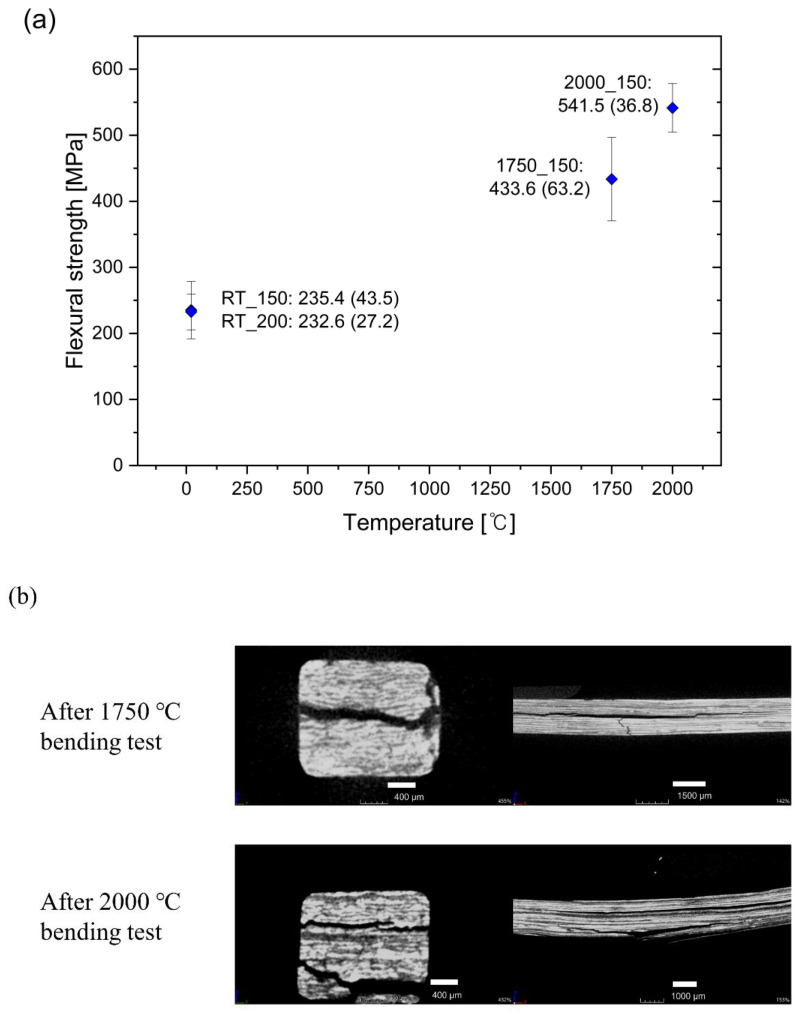

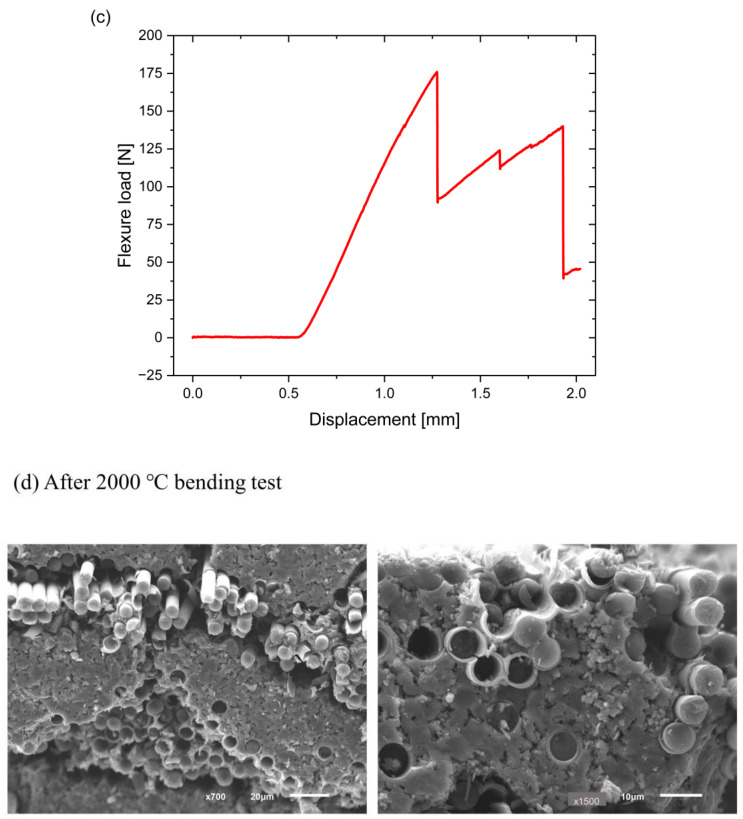
(**a**) Flexural strength of C_f_/SiC CMC at different temperatures. (**b**) Micro-CT images of C_f_/SiC CMC specimens after bending tests at 1750 °C and 2000 °C. (**c**) Load–displacement plot of C_f_/SiC CMC at 2000 °C. (**d**) SEM images from fractured surfaces of C_f_/SiC specimens after in situ bending test at 2000 °C.

Figure 3a summarizes the flexural strength of the C_f_/SiC CMCs measured at room temperature (RT), 1750 °C, and 2000 °C. The RT strengths of specimens prepared with different doctor blade gaps (150 or 200 μm) were similar (235 MPa with a standard deviation (SD) of 44 Mpa and 233 MPa with a SD of 27 MPa, respectively). Since the strengths were similar, the materials prepared with a doctor blade spacing of 150 μm were selected for further testing. Figure 3b shows microtomography images of fractured specimens after flexural testing at 1750 °C and 2000 °C. The images demonstrate that the specimens did not fracture into separate pieces and that brittle fracturing did not occur during the bending tests. Both crack deflection and delamination occur near the fracture surface. Crack deflection and delamination of the dense matrix could both function as important toughening mechanisms by deflecting propagating cracks toward the weak interfaces around the fiber and matrix discontinuities, respectively, that improve the fracture toughness and reliability of CMCs at room temperature [19,20]. Additionally, no large pores were observed with micro-CT, which confirmed that CMC fabrication suppressed defect formation in the matrix. Importantly, the flexural strength value at 1750 °C (434 MPa), which was 84% higher than at RT, further increased to 542 MPa at 2000 °C (Figure 3a).

The key reinforcement mechanism of a CMC is fiber pull-out. The successful function of this mechanism is essential to overcome the brittleness of SiC and improve its mechanical performance. A load–displacement curve from the test at 2000 °C is shown in Figure 3c. The curve shows that brittle fracturing did not occur during testing at 2000 °C based on the multiple-load drops, indicating effective fiber pull-out behavior. To confirm this, the cross section of the specimen fractured at 2000 °C was observed using SEM (Figure 3d). Carbon fibers with the pyrocarbon (PyC) interface exposed from the matrix were identified. The results clearly indicated that the SiC_p_/SiC PRC matrix manufactured by the present process was thermally stable up to 2000 °C and did not react with the PyC coating on the carbon fiber during thermal treatments or elevated temperature mechanical testing.

Two methods have generally been used to maintain the PyC weak interface on carbon fibers after densification with reactive matrices. The first method is to fabricate very thick PyC coatings. Nakazato et al. analyzed the optimum PyC coating thickness for matrix densification using hot pressing (NITE process) [21]. They reported that the thickness of the PyC coating decreased by approximately 400 nm during composite fabrication due to the reaction between the liquid phase produced by the sintering additive and the PyC interface. They concluded that the interface was damaged during the composite fabrication procedure, and therefore, the initial thickness of the PyC coating on the SiC fibers should be thicker than 500 nm to ensure quasi ductility of the NITE SiC/SiC composites.

The second method is to fabricate protective coatings on the PyC layer, such as PyC/SiC or PyC/BN/SiC [14,15]. Sing et al. applied single-layer pyrocarbon (PyC) coatings and multilayer PyC/SiC interfaces to the carbon fibers in C_f_/SiC CMCs when the matrix was processed by the I-CVI method. The fiber coatings were compared using flexural testing at 1200 °C in air, and they reported that the multilayer interface coating was beneficial compared to the single-layer coating for both flexural strength and damage tolerance because of the more tortuous and energy-absorbing crack path [14]. Additionally, the SiC layer on the surface of the multi-layer coating would provide better adhesion between fiber and matrix than the PyC or BN layer in direct contact with the SiC matrix.

SiC densified by the PIP method usually deteriorates at elevated temperature. Xu et al. reported a maximum strength of 235 MPa at 1200 °C under vacuum, with values that decreased from 1400 °C due to thermal deterioration [22]. Yin et al. reported that the strength of a SiC_f_/SiC CMC fabricated by PIP decreased to 13% of its room temperature value after heating in argon for 1 h at 1500 °C [23]. Lee et al. reported in 2009 that a C_f_/SiC_p_/SiBCN CMC fabricated by the PIP method retained 111% of its room temperature strength up to 1500 °C and 96% after heating to 1700 °C. The excellent thermal stability of the SiBCN PDC up to 2000 °C contributed to the excellent thermal stability of the CMC. However, SiBCN-based precursors are not commercially available and would be expensive and dangerous when considering the fabrication process [24]. Unlike previous reports, the present study found a continuous increase in the strength of the C_f_/SiC CMC up to 2000 °C despite using a commercial SiC precursor.

### 3.3. Mechanisms for the Increase in Elevated Temperature Strength

The thermomechanical stability of the matrix, which mostly consisted of nanosized SiC fillers, has the most important role in how the strength of the C_f_/SiC CMC manufactured by the PIP process could increase to 230% of the room temperature value at 2000 °C.

**Figure 4:**
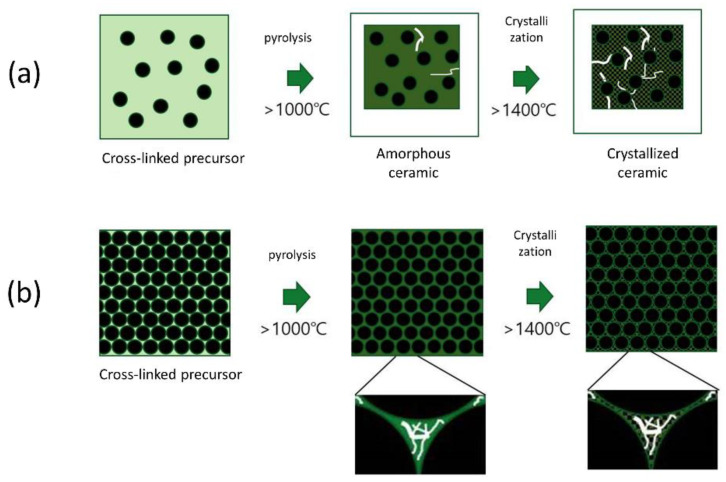
Scheme of mechanism showing the improvement of high-temperature properties of SiC_p_/SiC PRC matrix: (**a**) conventional process (filler: black circle) and (**b**) highly packed filler powder.

Figure 4 illustrates the possible mechanism regarding the thermal stability of the PRC matrix up to 2000 °C. Figure 4a illustrates the structure of the matrix made using the conventional PIP process. SiC slurries with less than 30 to 40 vol% solid loading have generally been used to fabricate CMCs. As a result, the lower volume fraction of the matrix consists of the SiC filler, while PDC occupies a majority of the volume. As the crystallization of amorphous SiC-based PDC progresses at temperatures over 1400 °C, the PDC volume may decrease by as much as 30% due to the increase in the density of the solid from ~2.2 g/cm^3^ to 3.2 g/cm^3^. Cracks that form in the PDC-SiC matrix under this condition cause deterioration of the mechanical properties of the PDC.

Figure 4b illustrates a matrix when filler particles occupy most of the volume, which can occur when using slurry with ultrahigh solid loading. In this case, the cracks that occur in the PDC-SiC are localized rather than connected with each other and do not strongly affect the strength of the specimen. Applied stress is supported by the network structure composed of the thermally stable SiC filler particles. Because sintering additives were not used, no liquid phase formed, which could also decrease the thermal and mechanical stability of SiC. Therefore, the matrix retained the superior properties of SiC at elevated temperature, and the C_f_/SiC CMC containing the thermally stable SiC-based matrix avoided deterioration of strength at temperatures up to 2000 °C.

**Figure 5:**
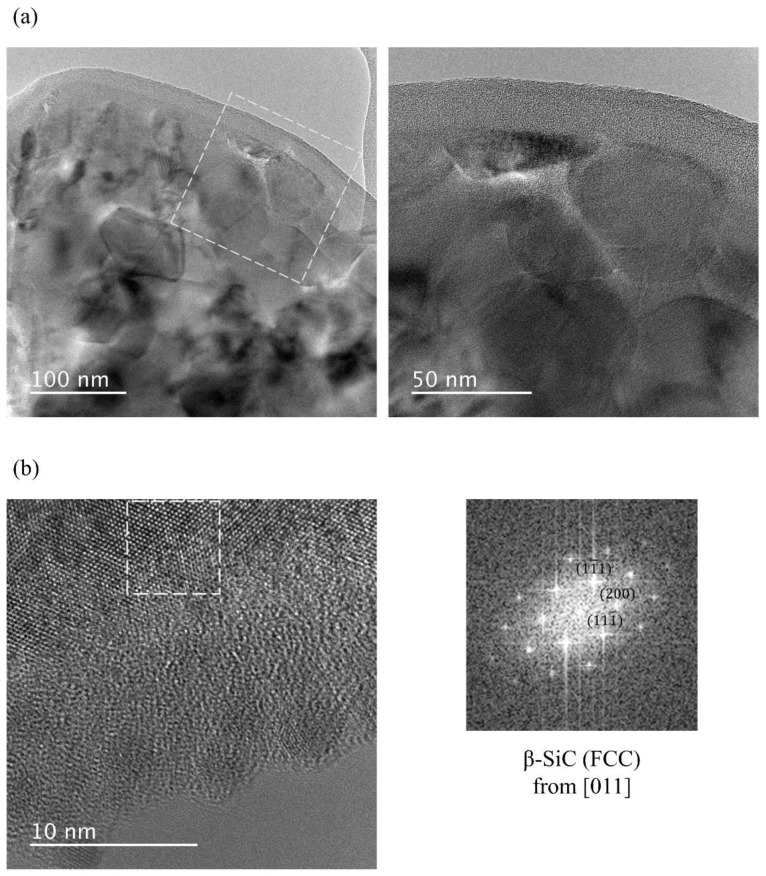
TEM images of the SiC_p_/SiC PRC matrix after heating to 2000 °C showing (**a**) crystallization and growth of nano-SiC crystallites within PDC, and (**b**) high-resolution image and its Fourier transform pattern.

Figure 5a shows the formation of PDC SiC and the morphology of fine grains grown to less than 100 nm in size within amorphous SiC after heating the SiC_p_/SiC PRC matrix to 2000 °C for 1 h under argon. Figure 5b indicates crystallization of β-SiC within the PDC, where the high-resolution image and fast Fourier transform (FFT) pattern are acquired from the dashed square in the image. Crystallization and growth of the grains have been reported to be the major factors in the reduction of strength at elevated temperatures for materials fabricated by PIP. Figure 5 indicates that crystallization and growth occurred in the present research. However, strength was retained up to at least 2000 °C. This indicates that the thermally stable and densely packed SiC filler was able to suppress crack growth and crack linking in the PDC. Additionally, our previous study reported a clear bond formation between SiC fillers and no sign of oxide segregation at the boundaries between PDC and SiC particles after heating to 2000 °C [13].

**Figure 6:**
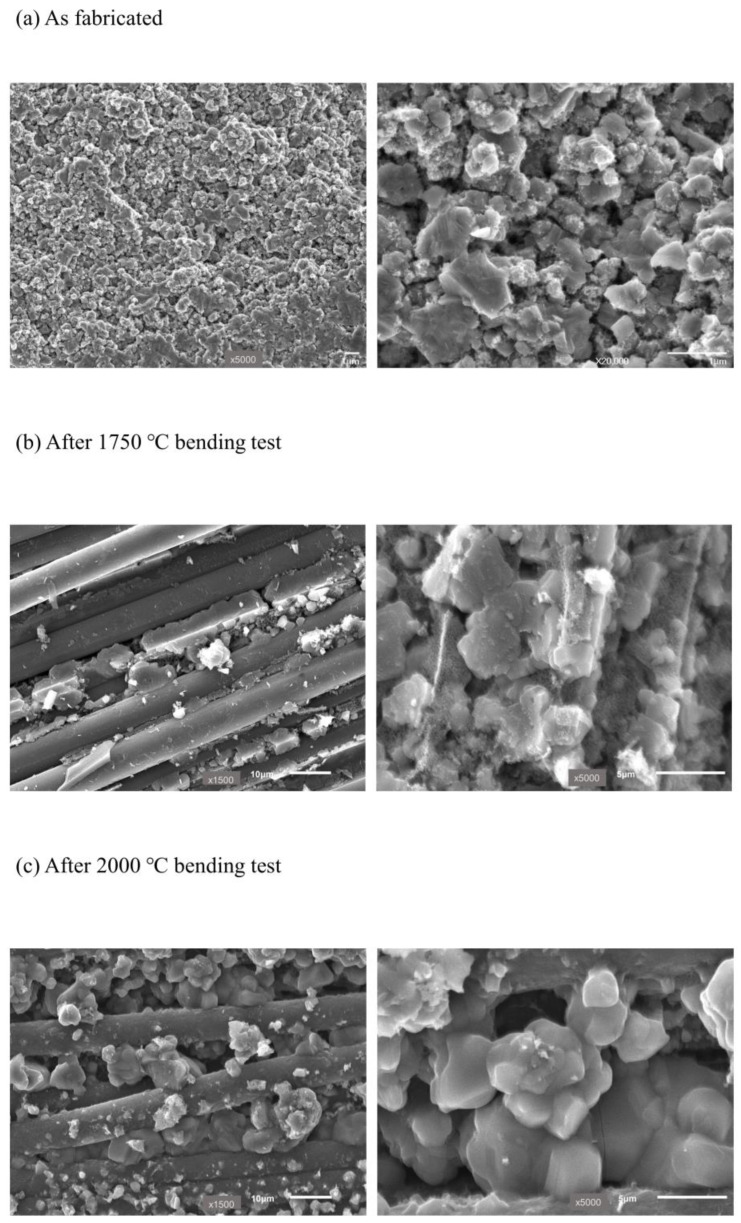
SEM images of C_f_/SiC CMC: (**a**) as-fabricated matrix, and after flexural testing at (**b**) 1750 °C and (**c**) 2000 °C.

The strength values increased at elevated temperatures based on the following two mechanisms. First, partial sintering of the matrix at a high temperature and blunting of sharp defects where stress could be concentrated lead to a decrease in the stress concentration. Pisch et al. reported that the evaporation of SiC occurred at 1900 °C, and the subsequent condensation induced the formation and growth of necks between SiC particles [25]. Figure 6 shows the microstructure of the matrix before and after the high-temperature test. Neck formation and growth of the fillers by partial sintering at an elevated temperature were observed. The surface area of the nano-SiC filler particles used in the present research decreased from 15.8 cm^2^/g to 10.4 cm^2^/g after heating at 1700 °C for 1 h, which also indicates the partial sintering of the SiC filler in this temperature range. Therefore, it is interpreted that during the partial sintering of the SiC_p_/SiC PRC matrix at elevated temperatures, any SiC that evaporated was likely to concentrate in sharp areas such as crack tips, which led to a blunting effect [2,26,27].

Second, partial sintering increased the bonding strength between SiC particles and/or between SiC particles and the PDC matrix, as shown in Figure 6b,c. This interpretation is supported by a previous study that reported an increase in hardness of a SiC_p_/SiC PRC manufactured using the same process as the present study of 46% and 61% after heat treatment at 1700 °C and 2000 °C, respectively [13].

The transformation from β-SiC to α-SiC at elevated temperatures may also affect the elevated temperature strength. In fact, after measuring the flexural strength at 1750 °C and 2000 °C, XRD analysis of the C_f_/SiC CMC revealed an increase in the α-SiC peak intensity (Figure 2b,c).

Table 1 summarizes the Rietveld refinement results of the XRD data that were presented in Figure 2. The SiC filler powder synthesized by mechanical alloying mostly consisted of β-SiC. At elevated temperatures, the proportion of α-SiC increased. The analysis showed that α-SiC started to form above 1700 °C, with further conversion at 2000 °C. However, even for the specimens heated to 2000 °C, the matrix phase still maintained over 80 vol% of β-SiC.

SiC and C_f_/SiC CMCs are potential candidates for elevated temperature applications in aircraft engines or aerospace structures, due to their desirable properties such as a low density and thermal expansion coefficient combined with excellent strength and wear resistance [10,14,22]. SiC-based CMCs have been successfully commercialized for aircraft engine parts, and the scope of application and production are rapidly expanding [5,6,7]. There are needs to develop processes to maintain or increase the properties while also reducing production time and costs. The present process uses an aqueous slurry and liquid precursor that enables more rapid manufacturing than conventional PIP processes. In addition, in spite of the relatively low processing temperature of 1500–1550 °C, SiC_p_/SiC PRC and C_f_/SiC CMCs exhibit excellent strength retention up to 2000 °C. Therefore, the present method appears to be an appropriate approach for the fabrication of SiC-based composites.

## 4. Conclusions

The elevated temperature strength of a C_f_/SiC CMC was analyzed. The materials were produced using an ultrahigh solid loading slurry prepared from a nano-SiC filler and densified through a high-speed PIP process. Compared to room temperature, the flexural strength increased by 84% at 1750 °C (235 MPa at RT vs. 434 MPa at 1750 °C) and increased further to 542 MPa at 2000 °C. The reasons for the excellent performance of the CMC are as follows: (1) the matrix forms a thermally stable SiC particle network structure, which becomes stronger at elevated temperatures by partial sintering; (2) as no sintering additive was used or residual silicon was formed during the PIP process, a harmful secondary phase did not form; (3) the essential CMC strengthening mechanism, ‘fiber pull-out’, successfully functioned; and (4) blunting of sharp edges and enhanced bonding was promoted by partial sintering. Because the process is based on a liquid phase route that uses liquid slurry and liquid precursor at a relatively low processing temperature below 1550 °C, the present results represent a possible way for the rapid production of CMCs with large, complex shapes and excellent performance.

## Figures and Tables

**Table 1:** Crystal structure parameters of the β-SiC (3C, F −4 3 m) and α-SiC (6H, P 63 m c) phases in the SiC_p_/SiC matrix analyzed by Rietveld refinement of the X-ray diffraction data.

	Parameter							
Specimen	Space Group	a = b [Å]	c [Å]	Volume [Å3]	Phase Fraction [vol%]	Rexp [%]	Rpro [%]	χ^2^
SiC_p_/SiC PRC	F −4 3 m	4.359	=a	82.800	100.0	7.91829	18.75110	7.53398
	P 63 m c	—	—	—	0.0			
SiC_p_/SiC PRC-1700C	F −4 3 m	4.355	=a	82.573	97.8	9.60238	17.39561	5.30152
	P 63 m c	3.095	15.245	126.484	1.2			
SiC_p_/SiC PRC-2000C	F −4 3 m	4.357	=a	82.720	82.2	10.35522	14.98942	3.15222
	P 63 m c	3.082	15.103	124.242	11.1			
C_f_/SiC CMC-1750C	F −4 3 m	4.359	=a	82.840	88.0	16.30605	39.98667	9.81793
	P 63 m c	3.080	15.136	124.325	12.0			
C_f_/SiC CMC-2000C	F −4 3 m	4.359	=a	82.839	81.2	13.85204	14.73738	2.27492
	P 63 m c	3.085	15.115	124.571	10.3			

## Data Availability

The data presented in this study are available on request from the corresponding author.
